# Fractal ventilation enhances respiratory sinus arrhythmia

**DOI:** 10.1186/1465-9921-6-41

**Published:** 2005-05-09

**Authors:** W Alan C Mutch, M Ruth Graham, Linda G Girling, John F Brewster

**Affiliations:** 1 Department of Anesthesia, Faculty of Medicine, Anesthesia Research Laboratory, University of Manitoba, Winnipeg MB, Canada; 2 Department of Statistics, Faculty of Science, University of Manitoba, Winnipeg MB, Canada

## Abstract

**Background:**

Programming a mechanical ventilator with a biologically variable or fractal breathing pattern (an example of 1/f noise) improves gas exchange and respiratory mechanics. Here we show that fractal ventilation increases respiratory sinus arrhythmia (RSA) – a mechanism known to improve ventilation/perfusion matching.

**Methods:**

Pigs were anaesthetised with propofol/ketamine, paralysed with doxacurium, and ventilated in either control mode (CV) or in fractal mode (FV) at baseline and then following infusion of oleic acid to result in lung injury.

**Results:**

Mean RSA and mean positive RSA were nearly double with FV, both at baseline and following oleic acid. At baseline, mean RSA = 18.6 msec with CV and 36.8 msec with FV (n = 10; p = 0.043); post oleic acid, mean RSA = 11.1 msec with CV and 21.8 msec with FV (n = 9, p = 0.028); at baseline, mean positive RSA = 20.8 msec with CV and 38.1 msec with FV (p = 0.047); post oleic acid, mean positive RSA = 13.2 msec with CV and 24.4 msec with FV (p = 0.026). Heart rate variability was also greater with FV. At baseline the coefficient of variation for heart rate was 2.2% during CV and 4.0% during FV. Following oleic acid the variation was 2.1 vs. 5.6% respectively.

**Conclusion:**

These findings suggest FV enhances physiological entrainment between respiratory, brain stem and cardiac nonlinear oscillators, further supporting the concept that RSA itself reflects cardiorespiratory interaction. In addition, these results provide another mechanism whereby FV may be superior to conventional CV.

## Background

Systems or computational biology – the use of mathematical analysis to examine complex biological systems – is becoming increasingly important [1,2]. Biological signals are complex, with fractal or even multi-fractal characteristics, and health is associated with fractal timing sequences [3]. For example, normal sinus rhythm is multi-fractal and the onset of congestive failure significantly attenuates this complex signal[4]. Respiratory sinus arrhythmia (RSA) – the increase in heart rate with inspiration and decrease with expiration – is one component of this complexity. It represents a dynamic interaction between respiratory, brain stem and cardiac oscillators that is physiologically advantageous. Hayano et al.[5]showed in dogs that positive RSA is associated with lower shunt fraction and lower dead space ventilation. Negative RSA – a decrease in heart rate with inspiration and increase with expiration – increases shunt fraction and dead space ventilation. Thus positive RSA improves ventilation/perfusion matching. Godin and Buchman[6] have proposed that the loss of RSA that occurs in illness is a consequence of uncoupling of important biological oscillators. Interventions that restore or enhance coupling would therefore be advantageous.

We have developed a so-called biologically variable mechanical ventilator, which uses a normal fractal-breathing pattern (a form of 1/f noise) to generate physiological rates and volumes. Long recordings of normal respiration are processed to drive the ventilator using computer hardware and software. In numerous models, fractal ventilation (FV) has been shown to improve gas exchange (increases arterial oxygen tension) and respiratory mechanics (more compliant lung and lower peak inspiratory pressure) with decreases in shunt fraction and dead space ventilation compared to conventional ventilation (CV) which delivers monotonously regular respiratory rates and tidal volumes [7-10]. Mechanisms for such improvement include increased surfactant phospholipid[11] and stochastic resonance – the addition of noise to an input signal to enhance an output in a nonlinear system[12]. We hypothesized that the imposition of a fractal respiratory signal with its added physiological noise could influence the cardiorespiratory oscillators and manifest as enhanced RSA. We measured RSA during FV and CV in pigs under control conditions and after oleic acid induced acute lung injury.

## Methods

**Figure 1:**
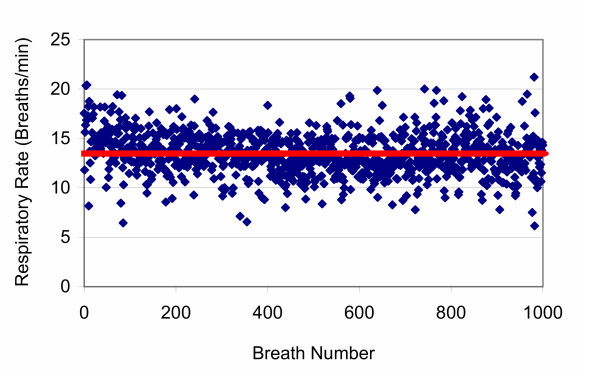
**Spontaneous human breathing pattern**. Breathing pattern used to program the fractal ventilator – data from awake human. Red line – mean rate.

**Figure 2:**
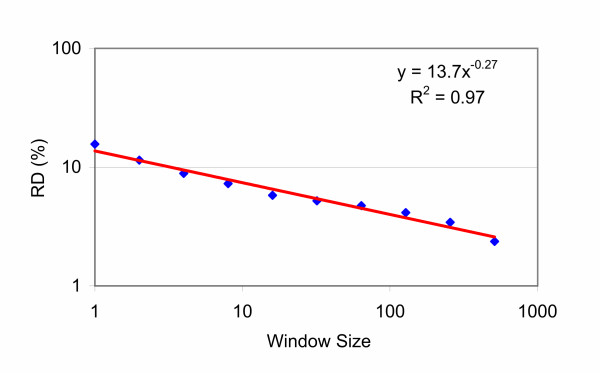
**Monofractal Analysis of the breathing pattern**. Monofractal or residual dispersion (RD) analysis – one of a number of ways that fractal behaviour can be analysed [25]. RD is defined as standard deviation/mean × 100. This analysis uses data from Figure 1 with pooled data (or window sizes) based on powers of 2^n ^following a log-log transform. A linear relationship indicates fractal behaviour, if the slope of the line lies between – 0.5 and 0. If these conditions are met the data fits an equation of the form y = 1/x^α ^defining a power law; with slope = α. The fractal dimension (D) is defined a 1 - α. D was 1.27 over 2.71 decades (log 512) with R^2 ^= 0.97. D of 1.0 defines a completely homogeneous data set, and 1.5 defines a completely random set (white noise). In between these two boundary conditions the data has fractal characteristics.

**Figure 3:**
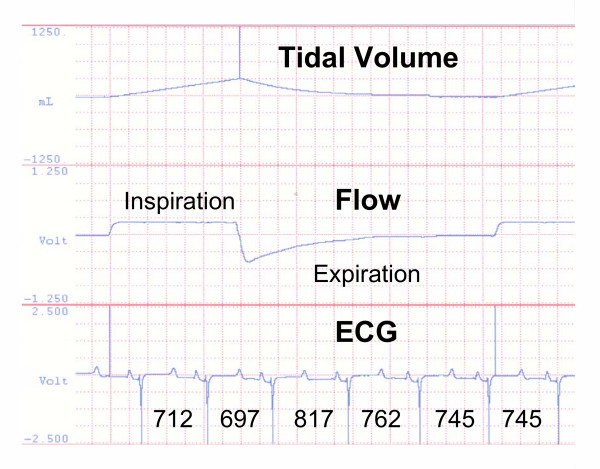
**Calculation of Respiratory Sinus Arrhythmia (RSA)**. Computer generated strip chart recording from data acquisition system showing how respiratory sinus arrhythmia (RSA) was determined during mechanical ventilation. Top panel – delivered tidal volume (millilitres), middle panel – inspiratory and expiratory flow, bottom panel – electrocardiogram (ECG). Below each heartbeat is the time in milliseconds (msec) of the beat interval. If the preceding beat was initiated in inspiration then this beat interval was considered to have occurred during inspiration and vice versa for expiration. In this respiratory cycle the RSA was positive at 120 msec (817 – 697 msec).

The Committee for Animal Experimentation at the University of Manitoba approved the study. The study was done in ten, 30–40 kg pigs. Pigs were sedated with midazolam (0.5 mg/kg)/ketamine (12 mg/kg)/atropine (0.6 mg) IM. When sedated, anaesthesia was induced with isoflurane 5% in oxygen by a tight fitting nose cone and the pig was intubated endotracheally. Anaesthesia was switched to a total intravenous technique using propofol (8 mg/kg/hr)/ketamine (2 mg/kg/hr) with doxacurium (10 mg/hr) for muscle relaxation. Arterial blood gases, airway pressures and respiratory system compliance (Crs) were determined at baseline. A Respironics Esprit^® ^ventilator was used for the duration of the experiment. The fractal-breathing file used to program the ventilator is shown in Figure 1. Data were obtained from an awake spontaneously breathing teenager sitting at rest. The mono-fractal analysis of the breathing file is shown in Figure 2. With FV the ventilator functioned as a volume divider – meaning the instantaneous minute ventilation product was constant. When rate was high, tidal volume was low and vice versa. Thus, minute ventilation was unchanged on converting from CV to FV or vice versa. The healthy animals were randomly allocated at recording period 1 to either FV or CV and then switched to the other mode for recording period 2. Simultaneous measurements of delivered tidal volume (ml), inspiratory and expiratory flow and electrocardiogram (ECG) were recorded at 400 samples/sec. RSA was determined as follows: the longest R-R interval in expiration was subtracted from the shortest R-R interval in inspiration (msec) – from data recorded to a digital acquisition system. If the difference in R-R interval was a positive value this was considered 'positive RSA'; if the difference determined had a negative value, then this was considered 'negative RSA'. If the preceding beat (R wave) was initiated in inspiration then this beat interval was considered to have occurred during inspiration and vice versa for expiration (Figure 3). Eighty to 100 breathing cycles were analysed in each ventilation mode. Mean respiratory rate was set to 20 breaths/min so data collection was over 4 – 5 min in each mode. The lungs were then injured by infusion of oleic acid intravenously to simulated acute respiratory distress syndrome. Blood gas, airway pressures and Crs were repeated. After injury, animals were randomly allocated to either of the two ventilation modes for recording period 3 and switched to the other mode for period 4.

## Results

No cross-over effects were seen for measurements of arterial blood gases, airway pressures or Crs between groups at either baseline or following infusion of oleic acid. One animal died following administration of oleic acid. Data were pooled for baseline and oleic acid time periods to demonstrate the nature of the lung injury. At baseline, over the measurement period, tidal volume was modestly higher with FV than CV; 225 mL versus 204 mL respectively (p = 0.039). Following lung injury, tidal volume did not differ; CV 188 mL versus 202 mL with FV (p = 0.26). When mean tidal volume was determined over a longer time interval (500 – 800 breath cycles), there were no differences between tidal volumes at baseline; CV, 203 ± 13 mL and FV, 208 ± 17 mL, or following oleic acid lung injury; CV, 188 ± 16 mL and FV, 185 ± 12 mL. At baseline PaO_2 _was 248 ± 22 mm Hg, and decreased to 121 ± 19 mm Hg following oleic acid infusion; PaCO_2 _was 43 ± 4 mm Hg and increased to 54 ± 6 mm Hg with oleic acid; Crs decreased from 1.00 ± 0.09 mL/cm H_2_O/kg to 0.55 ± 0.08 mL/cm H_2_O/kg after lung injury.

As calculated, RSA was usually positive by our definition. Mean RSA was the global average of both positive and negative determinations with each breathing cycle in a measurement period of 80 – 100 breaths. For further analysis, the incidence of mean positive RSA (RSA determined when only positive; presumed beneficial) and mean negative RSA (RSA determined when only negative; presumed detrimental) were examined. Mean RSA and mean positive RSA were nearly double with FV, both at baseline and following lung injury. At baseline, mean RSA = 18.6 msec with CV and 36.8 msec with FV (n = 10; p = 0.043); post oleic acid, mean RSA = 11.1 msec with CV and 21.8 msec with FV (n = 9; p = 0.028); at baseline, mean positive RSA = 20.8 msec with CV and 38.1 msec with FV (p = 0.047); post oleic acid, mean positive RSA = 13.2 msec with CV and 24.4 msec with FV (p = 0.026). The percent positive RSA at baseline was 88 ± 10% with CV and 95 ± 6% with FV (p = 0.051 between groups); following oleic acid injury the percent positive RSA moderately decreased in both groups to 79 ± 26% with CV and 84 ± 18% with FV (p = 0.079 between groups). Percent negative RSA was (100% – calculated positive RSA%) for each measurement period. At baseline the percent negative RSA was 12 ± 10% with CV and 5 ± 6% with FV (p = 0.051); following oleic acid injury the percent negative RSA was 21 ± 26% with CV and 16 ± 18% with FV (P = 0.079).

**Figure 4:**
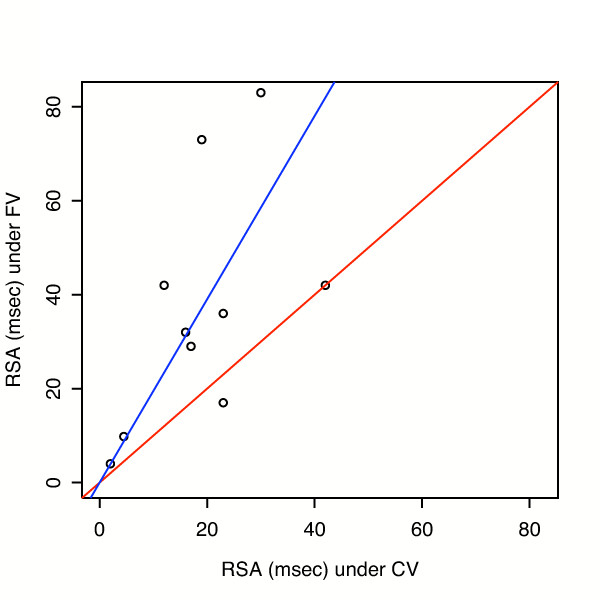
**RSA with Fractal Ventilation (FV) vs. RSA with Conventional Ventilation (CV) at baseline**. Regressions through the origin (blue line) were fit at baseline, using weighted least squares with weights proportional to 1/(RSA with CV). The estimated slope = 1.95 with standard error = 0.34; p = 0.010 for testing slope = 1 vs. slope >1. Red line has slope = 1.

**Figure 5:**
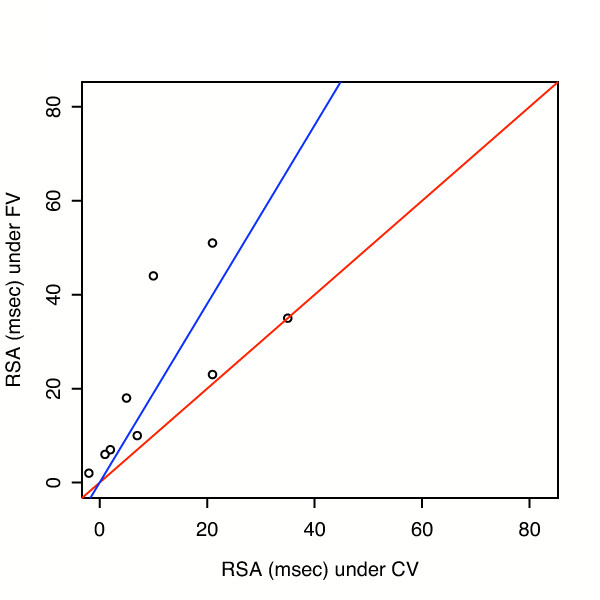
**RSA with Fractal Ventilation (FV) vs. RSA with Conventional Ventilation (CV) following oleic acid injury**. Regressions through the origin (blue line) post oleic acid injury, using weighted least squares with weights proportional to 1/(RSA with CV). Estimated slope = 1.90, standard error = 0.46 and p = 0.044. Red line is slope = 1.

The differences in mean RSA between groups is examined in more detail in Figure 4 and 5. In Figure 4, a regression through the origin (blue line) was fit to baseline data, using weighted least squares with weights proportional to 1/(RSA with CV). Estimated slope was 1.95 ± 0.34. FV points are above the red line of identity in 8 of 10 cases. In Figure 5, post oleic acid injury, estimated slope = 1.90 ± 0.46. We also analysed heart rate variability over more than 500 breaths. At baseline the coefficient of variation for heart rate was 2.2% during CV and 4.0% during FV. Following oleic acid the variation was 2.1 vs. 5.6% respectively.

## Discussion

RSA is a complex interaction between 3 nonlinear oscillators – the oscillatory pattern seen with breathing, the interface with discharge in the brain stem and the oscillation of the heartbeat. By flipping a switch on a mechanical ventilator to initiate a fractal-breathing pattern with its physiological or 1/f noise, an enhanced communication is evident between these three oscillators. A physiologically important entrainment has occurred. Hayano and Yasuma have advanced the hypothesis that RSA is an intrinsic resting function of the cardiorespiratory system[13]. In this broader context, RSA is postulated to "save cardiac and respiratory energy by suppressing unnecessary heartbeats during expiration and ineffective ventilation during waning phases of perfusion." By switching to FV, RSA has increased, thereby enhancing this intrinsic resting function of the cardiorespiratory system. This study shows that RSA decreases with severe lung injury following oleic lung injury, but fractal breathing remains associated with greater RSA. Heart rate variability is greater both at baseline and following lung injury with FV. Greater heart rate variability has been identified as an index of health [4,14].

Recent work by Amaral and colleagues[15] provides a mathematical model for why FV could enhance RSA in the context of emerging complex dynamics in signalling networks. They demonstrate that complex dynamics such as 1/f scaling of power spectra (as in fractal time sequences for heart rate or respiratory rate) can be generated when simple systems meet two requirements i) they manifest "small world" topology and ii) demonstrate noisy input. When signal input has these two characteristics and individual units in a network react to their input with physiological responses, the system demonstrates robustness to degradation. In their model, if long-range connectivity is low, model stability can be sustained by increased noise or vice versa. If the two conditions described by Amaral are met with FV, an important additional explanation is offered for how this noisy life support device may be an improvement over a conventional ventilator. The first condition is clearly met as we have added extraneous fractal noise with the computer-controlled variability file. The second condition is less obvious. However, the "small world" conditions for information transmission – the connections over long range – may be provided by the neural connections themselves. Such may be physically representative of the vagal afferents with lung stretch and efferents to the sinus node of the heart as linked through the brain stem where cardiorespiratory neurons are locally interconnected. As Amaral et al.[15] suggest, "the model may also provide a robust way to generate fluctuations that closely resemble physiological signals, which could be implemented in medical devices such as mechanical ventilators..." Programming a mechanical ventilator with normal physiological signals as with FV provides a means to generate the noisy fluctuations that in this circumstance enhances RSA; a complex dynamic between the respiratory, brain stem and cardiac oscillators. Thus, adding physiological noise with FV appears a working example of the emergence of complex dynamics as modelled mathematically[15].

Previous work has demonstrated that FV can improve gas exchange and respiratory mechanics by increased surfactant phospholipid[11] and through stochastic resonance – the addition of noise to an input signal to enhance an output in a nonlinear system[12]. Suki and colleagues[16] analysed the variable inflation pressures seen with FV and showed how this noisy signal can enhance recruitment of collapsed alveoli with their nonlinear opening characteristics. Findings in this study define a new mechanism whereby FV differs from conventional mechanical ventilation – by enhancing RSA – both in health and following lung injury to simulate acute respiratory distress syndrome.

Unlike the work by Hayano et al.[5], we did not measure shunt fraction, or dead space ventilation in these experiments. Changes in either variable were not expected given that the data measurement periods were limited to 4 – 5 minutes to determine RSA in each 30 min experimental period. Previous work from the laboratory suggests that advantageous changes in either dead space or shunt fraction occur over a minimum time course of 90 – 120 minutes following oleic acid injury with FV. However, this experiment showed that changes in RSA were measurable over a very short time period when switching between modes of ventilation.

The relationship between positive pressure ventilation and RSA is controversial [17-20]. Some studies indicate RSA remains intact while others show a reversal of the pattern. Importantly, Taha et al.[20] suggest that vagal feedback from pulmonary stretch receptors is obligatory for the generation of neurally mediated RSA. Positive RSA with spontaneous ventilation (negative pressure breathing) couples increased venous return to the inspiratory phase when oxygen tension is maximized. Increased venous return does not occur during inspiration with positive pressure ventilation. This change decouples augmented venous return and maximal oxygenation. However, the timing of the positive RSA in relation to where in the expiratory cycle the longest R-R interval occurs could still have physiological relevance. Thus, if the first beat in the expiratory cycle has the longest R-R interval, alveolar oxygen levels would be higher than if the positive RSA was associated with a beat later in the expiratory cycle. Such seems to be the case with our experimental conditions. By way of example, in 5 experiments during baseline measurement of RSA while in CV mode, there were only 2 heartbeats in each expiratory cycle over the measurement period comprising approximately 100 beats. Positive RSA would be recorded for a given R-R interval irregardless of the beat order if either of the two expiratory R-R intervals was greater than the inspiratory R-R interval, but in 82 ± 8 % of cases the longest R-R interval occurred with the first expiratory beat. As a further example, examination of Figure 3 during FV indicates that the greatest R-R interval occurs with the first expiratory heartbeat when 4 heartbeats are initiated within this expiratory cycle.

Anaesthesia, both type and depth can influence RSA [21,22]. However, the depth of anaesthesia was a controlled variable in our experiment as we employed a continuous infusion of propofol/ketamine with a cross-over design, thereby minimizing time effects.

More elegant means to measure RSA have been published. One such example is from recent work by Giardino et al.[23], which describes a sophisticated spectral analysis for determination of RSA in human subjects. They had patients breath rhythmically by following a respiratory-pacing stimulus displayed on a computer monitor. If for example, the patient was breathing at 12 breaths/min, RSA was measured as the amplitude of a 0.20-Hz sine wave fitted to the heart rate series. Such an analysis could be undertaken to assess RSA with CV where breathing frequency is monotonous, but not for FV where the breathing rate is variable over time. Thus, we chose a simpler approach to determine RSA.

## Conclusion

Our findings may have important implications for the design of life support devices used clinically[3]. Buchman has suggested that multiple organ dysfunction, often seen in patients requiring life support in the intensive care unit, is a consequence of lost coupling between "communicating" organ systems[24]. The inability to re-couple following the perturbation of critical illness may be an important reason why multi-organ system dysfunction is so lethal[6]. Fractal ventilation, by augmenting respiratory sinus arrhythmia, may be one approach to enhance organ system re-coupling.

## Competing interests

Dr. Mutch is co-founder of Biovar Life Support Inc., which has developed the mechanical ventilator described in this paper. Worldwide exclusive rights to this ventilator have been licensed to Respironics Inc. To date no ventilators have been sold clinically. In the event of sales of this ventilator, Dr. Mutch and the University of Manitoba would stand to gain financially. None of the other authors have a financial interest in the ventilator.

## Authors' contributions

WACM conceived the study, helped analyse and interpret the data and helped write the paper. MRG helped conduct the study, analyse and interpret the data and write the paper. LGG conducted the study, collated data, and helped prepare the figures. JFB analysed the data, conducted the statistical analysis, helped prepare the figures and helped write the paper.
